# Treatment outcomes and post-treatment mortality among people with multidrug/rifampicin-resistant tuberculosis in Sierra Leone: a national cohort study

**DOI:** 10.1016/j.lanafr.2026.100061

**Published:** 2026-10

**Authors:** Faye Greenwood, Rashidatu Fouad Kamara, Ronnie Harding, Ousman Conteh, Lynda Foray, Matthew J Saunders, Tom Wingfield

**Affiliations:** a Liverpool School of Tropical Medicine, Liverpool, UK; b Ministry of Health and Sanitation, Freetown, Sierra Leone; c Lakka Hospital, Freetown, Sierra Leone; d Njala University, Freetown, Sierra Leone; e Institute for Infection and Immunity, City St George's, University of London, London, UK; f Faculty of Public Health and Policy, London School of Hygiene & Tropical Medicine, London, UK; g Centre for Tuberculosis Research, Department of Clinical Sciences and International Public Health, Liverpool School of Tropical Medicine, Liverpool, UK; h Tropical and Infectious Diseases Unit, University Hospitals Liverpool, Liverpool, UK; i Department of Global Public Health, Karolinska Institutet, Stockholm, Sweden

**Keywords:** Tuberculosis, Multidrug-resistant, Rifampicin-resistant, Treatment outcome, Post-TB, Risk factors, Sierra Leone

## Abstract

**Background:**

Evidence on biosocial factors driving adverse outcomes in multidrug/rifampicin-resistant tuberculosis (MDR/RR-TB) can inform design of person-centred interventions. We evaluated treatment outcomes, post-treatment mortality, and their associated risk factors among people with multidrug/rifampicin-resistant tuberculosis (MDR/RR-TB) in Sierra Leone.

**Methods:**

We did a national cohort study of people with MDR/RR-TB in Lakka Hospital, Freetown, 2017–2022. Treatment was with WHO-recommended short (9-11 month) or long (18-24 month) regimens, with adverse treatment outcome (death or loss to follow-up before or during treatment, or treatment failure) following WHO definitions. Participants with treatment success had 12-months follow-up to ascertain post-treatment mortality. We used directed acyclical graph-informed logistic regression to investigate biosocial factors associated with adverse treatment outcome and post-treatment mortality.

**Findings:**

We recruited 739 people with MDR/RR-TB, median age 34-years (IQR: 25–45), 70% (517/739) male and 22% (160/734) HIV-positive. 22% (161/739) had adverse treatment outcome, with 18% (134/739) dying before or during treatment. Adverse treatment outcome was associated with age ≥45-years versus 25–44-years (adjusted odds ratio [aOR] = 1.9; 95% confidence interval: 1.1–3.2); unemployment (aOR = 2.9; 1.8–4.8); HIV (aOR = 1.8; 1.1–2.7); chronic lung disease (aOR = 1.7; 1.0–2.9); renal impairment (aOR = 4.8; 1.8–12.9); underweight (aOR = 1.6; 1.1–2.4); and long regimen (aOR = 2.0; 1.3–3.2). Among those with treatment success, 9.9% (57/578) had post-treatment mortality. This was associated with HIV (aOR = 2.0, 1.0–3.9), smoking (aOR = 2.7, 1.3–5.5), chronic lung disease (aOR = 2.2, 1.1–4.7), previous loss-to-follow-up (aOR = 3.1, 1.1–8.7) and severe TB on their original chest radiography (aOR = 2.4, 1.2–4.5).

**Interpretation:**

Adverse outcomes, including death after treatment success, were common among people with MDR/RR-TB in Sierra Leone and associated with modifiable risk factors, highlighting the urgent need for holistic, person-centred biosocial interventions for people with MDR/RR-TB throughout and beyond treatment.

**Funding:**

Medical Research Council-MR/Y503216/1; Medical Research Foundation-MRF-131-0006-RG-KHOS-C0942.

## Abstract (es)

**Antecedentes:**

La evidencia sobre factores biosociales que determinan resultados adversos en tuberculosis multirresistente/resistente a rifampicina (TB-MDR/RR) puede informar intervenciones centradas en la persona. Evaluamos los resultados del tratamiento, la mortalidad postratamiento y sus factores asociados en Sierra Leona.

**Métodos:**

Realizamos un estudio de cohorte nacional en personas con TB-MDR/RR atendidas en el Hospital Lakka, Freetown, entre 2017 y 2022. El tratamiento siguió regímenes cortos (9–11 meses) o largos (18–24 meses) recomendados por la OMS. Los participantes con éxito terapéutico fueron seguidos durante 12 meses para evaluar mortalidad postratamiento. Se utilizó regresión logística informada por grafos acíclicos dirigidos para analizar factores biosociales asociados con resultados adversos y mortalidad postratamiento.

**Resultados:**

Se incluyeron 739 participantes (edad mediana 34 años [RIC 25–45]; 70% hombres; 22% VIH positivos). El 22% (161/739) presentó resultados adversos, incluyendo 18 % (134/739) de muertes antes o durante el tratamiento. Los factores asociados incluyeron edad ≥45 años (aOR=1.9; IC95% 1.1–3.2), desempleo (2.9; 1.8–4.8), VIH (1.8; 1.1–2.7), enfermedad pulmonar crónica (1.7; 1.0–2.9), insuficiencia renal (4.8; 1.8–12.9), bajo peso (1.6; 1.1–2.4) y régimen largo (2.0; 1.3–3.2). Entre quienes lograron éxito terapéutico, 9.9 % (57/578) fallecieron tras el tratamiento, asociado con VIH, tabaquismo, enfermedad pulmonar crónica, pérdida previa de seguimiento y TB grave en radiografía.

**Interpretación:**

Los resultados adversos, incluida la mortalidad tras el éxito terapéutico, son frecuentes y se asocian con factores modificables, lo que resalta la necesidad urgente de intervenciones integrales centradas en la persona más allá del tratamiento.

**Financiación:**

Medical Research Council; Medical Research Foundation.

## Résumé

**Contexte:**

Les données sur les facteurs biosociaux associés aux issues défavorables de la tuberculose multirésistante/résistante à la rifampicine (TB-MR/RR) peuvent orienter la conception d’interventions centrées sur la personne. Nous avons évalué les issues du traitement, la mortalité post-traitement et leurs facteurs de risque chez des personnes atteintes de TB-MR/RR en Sierra Leone.

**Méthodes:**

Nous avons réalisé une étude de cohorte nationale incluant des personnes atteintes de TB-MR/RR prises en charge à l’hôpital de Lakka, Freetown, entre 2017 et 2022. Les traitements suivaient les schémas courts (9–11 mois) ou longs (18–24 mois) recommandés par l’OMS. Les participants ayant obtenu un succès thérapeutique ont été suivis pendant 12 mois afin d’évaluer la mortalité post-traitement. Une régression logistique informée par des graphes acycliques dirigés a été utilisée pour analyser les facteurs biosociaux associés aux issues défavorables et à la mortalité post-traitement.

**Résultats:**

Au total, 739 personnes ont été incluses (âge médian 34 ans [IQR 25–45]; 70 % d’hommes; 22 % vivant avec le VIH). Une issue défavorable a été observée chez 22 % (161/739), dont 18 % (134/739) de décès avant ou pendant le traitement. Les facteurs associés comprenaient : âge ≥45 ans (aOR=1,9; IC95 % 1.1–3.2), chômage (2.9; 1.8–4.8), VIH (1.8; 1.1–2.7), maladie pulmonaire chronique (1.7; 1.0–2.9), insuffisance rénale (4.8; 1.8–12.9), insuffisance pondérale (1.6; 1.1–2.4) et schéma long (2.0; 1.3–3.2). Parmi les patients en succès thérapeutique, 9.9 % (57/578) sont décédés après traitement, associés au VIH, tabagisme, maladie pulmonaire chronique, antécédent de perte de suivi et TB sévère à la radiographie.

**Interprétation:**

Les issues défavorables, y compris après succès thérapeutique, sont fréquentes et liées à des facteurs modifiables, soulignant la nécessité urgente d’interventions globales centrées sur la personne au-delà du traitement.

**Research in context**

**Evidence before this study**

In previous research, we described treatment outcomes and identified risk factors for adverse outcomes among a cohort of 365 people with multidrug/rifampicin-resistant tuberculosis (MDR/RR-TB) in Sierra Leone between 2017 and 2019. We demonstrated that adverse outcomes in this population were common and associated with several biomedical and social risk factors, and argued for the importance of integrating interventions to address these risk factors alongside improved clinical management of MDR/RR-TB.

For this study, we searched PubMed for studies published since our previous study in 2022 until 4th October 2025 using the terms (“TB” or “tubercul∗”) AND (“Multi-drug” or “multidrug” or “MDR” or “rifampicin-resistant” or “rifampicin resistant” or “RR”) AND (“outcomes” or “treatment outcomes” or “adverse outcome”). Our search returned 894 studies undertaken in a range of settings and populations spanning observational studies describing treatment outcomes and risk factors for adverse outcomes, to clinical trials assessing new treatment regimens for MDR/RR-TB. We undertook a narrative synthesis of this evidence to inform the development of a directed acyclic graph illustrating the pathways through which we hypothesised a range of demographic and social (e.g. age), health-related (e.g. HIV), and disease-related (e.g. disease severity) factors that might influence the risk of adverse outcomes in people with MDR/RR-TB.

Of note, we also found one study from Vietnam published in 2025, which demonstrated a high mortality rate among people with MDR/RR-TB extending for several years following apparent successful treatment. This finding supports a 2019 systematic review, which found high long-term all-cause mortality among people with TB compared with the general population or matched controls. Since conducting the original search above, a 2026 nation-wide Brazilian cohort study has also suggested increased long-term risk of death from any cause for up to 14 years following TB and TB treatment.

**Added value of this study**

This study builds substantially on our previous research. It is the largest single-country cohort study describing treatment outcomes among people with MDR/RR-TB in West Africa. In addition, to our knowledge, it is the first study from Africa to describe mortality following treatment success in this population and identify risk factors for adverse outcomes both during treatment and following treatment success.

Among a cohort of 739 people with MDR/RR-TB in Sierra Leone recruited until the end of 2022 and followed until September 2024, we found that over a fifth had an adverse treatment outcome, driven by deaths occurring both before and during treatment. These adverse treatment outcomes were associated with a range of biomedical and social risk factors, including older age, unemployment, HIV, chronic lung disease, renal impairment, other comorbidities, underweight, specific abnormalities on chest radiography, and receiving a long treatment regimen.

Then, even among people with MDR/RR-TB who had apparent treatment success, we found that one in 10 died in the subsequent 12 months. This high post-treatment mortality was associated with HIV, smoking, chronic lung disease, previous loss to follow-up on TB treatment, and severe TB on chest radiography.

**Implications of all the available evidence**

The available evidence demonstrates that MDR/RR-TB continues to threaten efforts to end the global TB epidemic. The high post-treatment mortality observed in our cohort suggests that the burden of ill health caused by MDR/RR-TB is likely to be substantially underestimated by programmatic data on treatment outcomes routinely reported by national TB programmes and the World Health Organization (WHO).

Our findings demonstrate the urgent need to develop and evaluate holistic, person-centred biosocial interventions to improve outcomes among people with MDR/RR-TB globally. Alongside improved tests and treatments, these interventions should consider the broader biomedical and social risk factors driving adverse outcomes, and extend through and beyond the immediate treatment period. Such interventions could accelerate progress in TB care and prevention while yielding substantial wider social and health benefits for people with MDR/RR-TB and their households.

## Introduction

Tuberculosis (TB) is the leading cause of death from a single infection globally, killing 1.25 million people in 2023.^1^ Approximately 400,000 people were estimated to have multidrug/rifampicin-resistant TB (MDR/RR-TB) in 2023, defined as TB resistant to rifampicin with or without additional resistance to isoniazid, and less than half of these were diagnosed and enrolled on treatment.^1^ Multiple factors drive development of drug resistance including diagnostic error, lack of drug susceptibility testing (DST), inappropriate or ineffective treatment regimens, treatment interruptions, and reduced adherence.^2^ Poor treatment outcomes for drug-sensitive TB (DS-TB) coupled with increasing community transmission of resistant strains are significant contributors to MDR/RR-TB emergence and circulation.^3^

Whilst curable, MDR/RR-TB treatment regimens have historically been longer, more costly to both people with TB and health systems, and associated with greater adverse effects than DS-TB treatment regimens.^4^^,^^5^ This results in worse treatment outcomes among people with MDR/RR-TB globally (71% had treatment success versus 88% for DS-TB in 2022, the last year with data available),^1^ and increased risk of TB recurrence with associated negative long-term health, psychological, and socioeconomic consequences.^6^ Importantly, emerging evidence has highlighted that people with TB, and particularly MDR/RR-TB, have a higher mortality rate than the general population even after apparent treatment success.^7^, ^8^, ^9^ There is limited evidence available, however, on the biomedical and social risk factors driving this phenomenon.^10^

Sierra Leone is a low-income country designated by World Health Organization (WHO) as one of 30 high TB burden countries globally with a TB incidence of 283/100,000 people in 2023.^1^ Only 42% of people with a new episode of bacteriologically confirmed pulmonary TB were estimated to be tested for rifampicin resistance in 2023. In 2020, the last year with data available, only 73% of people who were enrolled on treatment for MDR/RR-TB were recorded as having treatment success–far short of WHO's 2015 End TB Strategy targets.^1^

In previous research, we described treatment outcomes and identified risk factors for adverse outcomes among 365 people with MDR/RR-TB in Sierra Leone.^11^ In the current study, we extended this research to describe both treatment outcomes and death after treatment success (post-treatment mortality) among a much larger cohort, over twice the size of our previous research. Furthermore, we aimed to use more rigorous methodology to characterise how a range of biomedical and social risk factors influence the risk of adverse outcomes to support the design of holistic, person-centred biosocial interventions to address the needs of people with MDR/RR-TB.

## Methods

### Study design, setting, and participants

We did a national, retrospective cohort study of people with MDR/RR-TB notified to the National Leprosy and TB Control Programme (NLTCP) in Sierra Leone. Diagnosis of MDR/RR-TB in Sierra Leone is typically through Xpert MTB/RIF on sputum, with subsequent phenotypic drug susceptibility testing (DST) for people with rifampicin resistance supported by a supra-national reference laboratory in Borstel, Germany (Appendix, p2). A very small minority (<5%) are diagnosed with MDR/RR-TB on the basis of clinical signs and symptoms (e.g. based upon poor treatment response to first-line treatment, but with no microbiological evidence of rifampicin resistance).^11^

Upon diagnosis, in line with national guidelines, all people with MDR/RR-TB are referred for inpatient hospital management (irrespective of symptoms) in one of three government supported treatment centres: Lakka, Kono or Makeni.^12^ Until 2020, Lakka Hospital in Freetown oversaw the management of all people with MDR/RR-TB in Sierra Leone. Although management has been expanded to other regional centres, Lakka Hospital remains responsible for the care of the vast majority of people with MDR/RR-TB nationally.

In this cohort study we included people of any age who had bacteriologically confirmed MDR/RR-TB and were managed at Lakka Hospital between 17th April 2017 and 25th December 2022. People with no available demographic data (age and sex), and those transferred to another treatment centre or found to be misdiagnosed, were excluded.

### Procedures

All people included in the cohort were managed within the Lakka Hospital NLTCP programme. Participants were identified by NLTCP staff using nationally developed NLTCP registers and invited to give their informed verbal consent to analyse routinely collected, deidentified data obtained during hospital admission. Consistent with NLTCP procedures for routine collection of programmatic data with no additional data collection activities, procedures specific to this study, or potential risk to participants, written informed consent was not obtained.

Pharmacological treatment of MDR/RR-TB with short or long regimens was at the discretion of attending clinical NLTCP physicians, guided by individual history, clinical characteristics (e.g. disease severity, previous drug exposures, and results of DST), and response to treatment (e.g. adverse drug reactions, sputum smear/culture conversion).^12^ During the data collection period, MDR/RR-TB treatment guidance in Sierra Leone transitioned. Between 2016 and 2019, in line with WHO guidance at the time, bedaquiline was used on an individualised basis according to key criteria, such as intolerance or side effects to aminoglycosides.^13^ From 2020 onwards, bedaquiline use was no longer individualised and it was introduced routinely into treatment regimens. Changes to the long regimen, particularly, were considerable, moving from an injectable-containing regimen to an all-oral regimen (Appendix p3).

Participants were discharged from Lakka Hospital on the basis of sputum smear/culture conversion at the discretion of the attending clinical NLTCP physicians after approximately four to six months to complete ambulatory treatment in the community, typically for a further five or 12 months for people receiving short or long regimens respectively. Where possible, this was supported by directly observed therapy involving daily visits by NLTCP nurses and a transport allowance provided to both nurses and people with MDR/RR-TB to support treatment adherence. Following treatment success (cured or treatment completed, Appendix p4), participants were followed for 12 months to ascertain post-treatment mortality. Here, as part of routine NLTCP activities, NLTCP nurses supported by a stipend, transport costs, and mobile phone top-up charges conducted monthly phone calls to participants or their household members, and/or home visits when phone calls were not possible, to ascertain whether people were alive or known to have died. Follow-up continued until 5th September 2024 when funding for this activity ceased.

### Outcomes

We considered two outcomes, adverse treatment outcome and post-treatment mortality. Adverse treatment outcome was defined in line with national guidance and WHO definitions as a composite of death or loss to follow-up before or during treatment, or treatment failure (Appendix, p4).^14^ Treatment outcomes were assigned by NLTCP staff and ascertained from treatment registers and clinical notes. For people who started on a short regimen and switched to a long regimen because of treatment failure, we recorded their final treatment outcome on a long regimen. Post-treatment mortality was defined as known death in the 12 months following treatment success, ascertained through the follow-up described above (the denominator was therefore by definition only people with MDR/RR-TB who had treatment success, excluding those with a previous adverse treatment outcome). We did not have data available on timing for either outcome, timing of follow-up phone calls, causes of death, or on TB recurrence after treatment success.

### Risk factors and theoretical framework

Routinely collected baseline data at diagnosis and hospital admission were broadly categorised into three risk factor domains.

- *Demographic and social risk factors* included age (categorised as 0–24 years, 25–44 years, and 45 years and over for analysis as in our previous research),^11^ sex, district residence in Sierra Leone, history of incarceration, and whether people were employed or not.
- *Health-related risk factors* included smoking, HIV (with testing offered to all those whose HIV status was unknown or previously negative), diabetes (self/physician-reported), renal impairment (self/physician-reported), other comorbidities (self/physician-reported), previous TB (defined in alignment with WHO definitions, Appendix p5), and chronic lung disease (presence of chronic obstructive pulmonary disease, fibrosis, or bronchiectasis on chest radiography reported by radiologists at Lakka Hospital).
- *Disease-related risk factors* included mycobacterial culture result, drug resistance (ascertained through phenotypic DST for several second-line drugs), sputum smear microscopy grade, chest radiography abnormalities (including severe TB defined as the presence of cavitations or a miliary pattern), body mass index (BMI in kg/m^2^, categorised as in our previous research), and treatment regimen (“short” only versus “long”, with people who were switched from a short to a long regimen during treatment included in the long category).^11^

The study was undertaken explicitly from social epidemiologic perspectives of disease distribution.^15^ We drew a directed acyclic graph (DAG), informed by a rigorous literature review, to illustrate the pathways through which we hypothesised upstream demographic and social risk factors influenced downstream health-related risk factors, disease-related risk factors, and finally adverse outcomes (both adverse treatment outcome and post-treatment mortality) (Fig. 1).^16^ Within each domain of risk factors (illustrated as a “super-node” in the DAG), we also hypothesised a hierarchy, e.g. within the demographic and social risk factors we hypothesised that age influenced risk of incarceration and occupation.

**Fig. 1:**
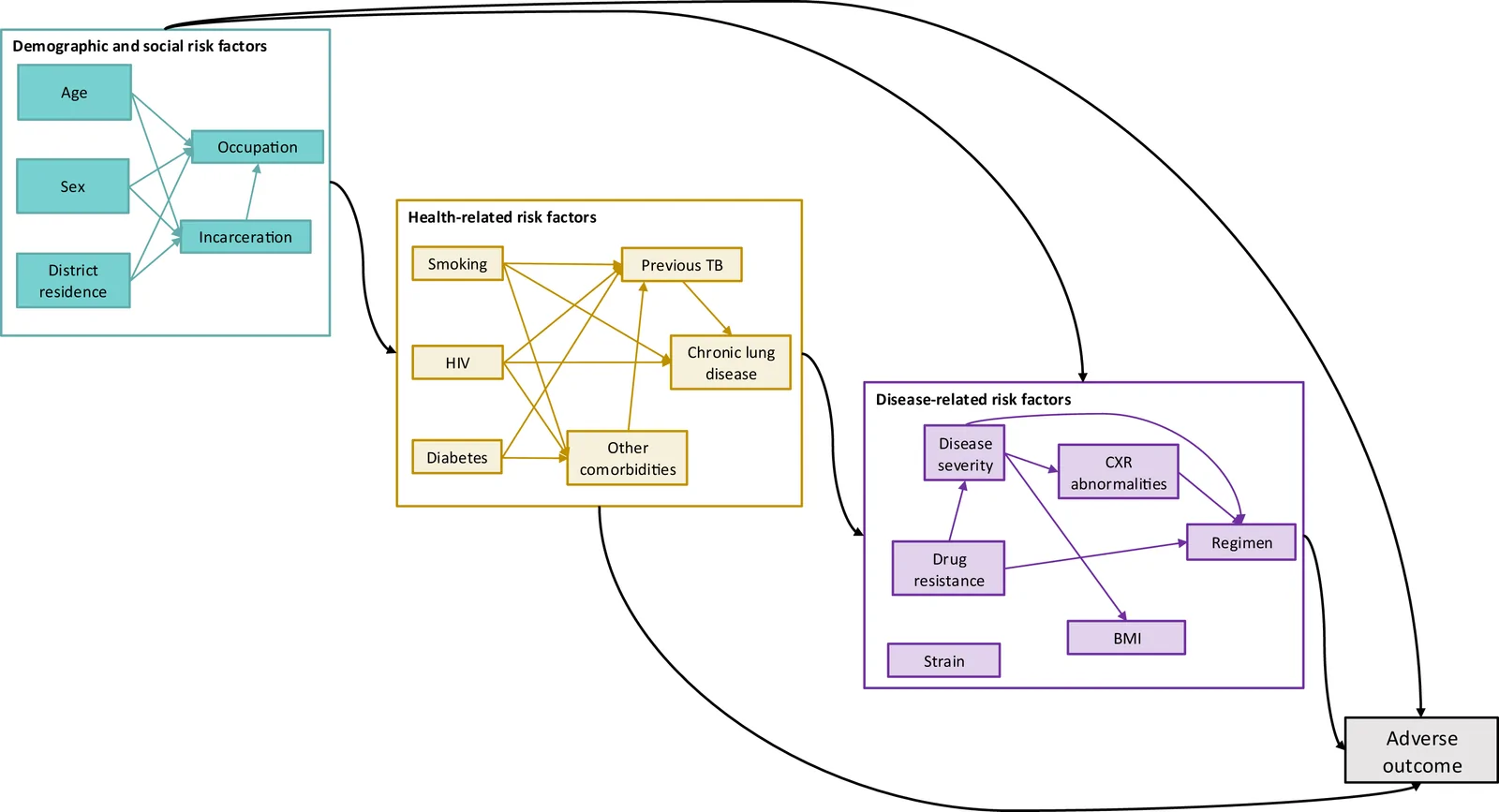
Simplified directed acyclic graph (DAG) illustrating hypothesised causal relationships between demographic and social, health-related, and disease-related risk factors and adverse outcome (adverse treatment outcome or post-treatment mortality) in people with MDR/RR-TB. MDR/RR-TB, multidrug/rifampicin-resistant tuberculosis. Solid thick arrows indicate that all variables in the upstream node (illustrated as boxes) were hypothesised to potentially influence all variables in the downstream node (e.g. all demographic and social risk factors were hypothesised to potentially influence all health-related risk factors and all disease-related risk factors, as well as influencing the risk of adverse outcome through other unmeasured pathways). Other comorbidities encompass renal impairment and other diseases self-reported by participants and/or documented by healthcare workers in participants' routine medical records. Disease severity includes sputum smear grade, assuming higher smear grades reflect more severe disease, and chest radiography findings of cavitations or miliary TB, again assuming these radiological findings to reflect more severe disease. Drug resistance refers to a collection of specific variables defining resistance to individual drugs. CXR abnormalities refer to a collection of specific variables (consolidation, pneumothorax, effusion, lymphadenopathy, and nodules) detected through chest radiography. Strain is determined through mycobacterial culture. See Table 1 for all variables under study. Boxes around individual variables are a formatting choice and not an indication of adjustment.

### Statistical analysis

All analyses were performed using Stata (version 18, StataCorp) and RStudio (version 2023.03.1 + 446). Continuous variables were summarised by their medians and interquartile ranges (IQR), while binary and categorical variables were summarised as proportions. Although the number of participants with missing data was small (<5%) for nearly all variables, we used multiple imputation with chained equations to replace missing values, assuming data were missing at random. Ten imputations were generated, and Rubin's rules were used to combine estimates for the analysis across the imputed datasets.^17^

Because we did not have data available on timing of the outcomes, we described the simple proportion of participants with each outcome. To maximise use of data, participants who were known to be less than 12 months following treatment success at the end of the study were included in the denominator for post-treatment mortality, with death ascertained up until their last follow-up phone call or household visit.

We used logistic regression to investigate the association between risk factors and both outcomes, calculating adjusted odds ratios (aOR) and 95% confidence intervals (95% CI). Rather than building a single multivariable model for each outcome including all risk factors (as in our previous research),^11^ we built separate models for each individual risk factor for each outcome with minimum adjustment sets for confounders included in each model based on our DAG (Appendix, p6). This process enables explicit illustration of our causal assumptions, and reduces the risk of biases and overadjustment for potential mediators between the risk factor of interest, and the outcome.^16^^,^^18^ Because this was hypothesis driven, we did not perform a statistical correction for multiple hypothesis testing.^19^

Additionally, to assess the population-level impact of specific risk factors on adverse treatment outcomes and inform the design of potential interventions, we calculated the population attributable fraction (PAF) of adverse treatment outcomes attributable to key potentially modifiable risk factors that demonstrated evidence of an association (defined by an odds ratio with a lower 95% CI of 1.0 or over or higher 95% CI of below 1.0). We did this using the punaf function in Stata.^6^

### Ethics

Ethical approval was obtained from the Sierra Leone Ethics Review and Scientific Committee (SLERSC) of the Ministry of Health and Sanitation, Government of Sierra Leone (SLERSC No: 015/11/2023) with Liverpool School of Tropical Medicine (LSTM) Research Ethics Committee review and ethic exemption issued. The study was endorsed by the Sierra Leone NLTCP.

## Results

### Cohort profile

753 people were notified with MDR/RR-TB in Sierra Leone during the study period, of whom 99% (747/753) were managed at Lakka Hospital. Of these, 99% (739/747) were included in our study (Fig. 2). 99% (738/739) had a positive Xpert MTB/RIF result, of whom 98% (722/738) had rifampicin resistance. The median age was 34 years (IQR: 25–45 years), 70% (517/739) were male, 22% (160/734) were HIV positive, and 60% (439/729) were underweight. Detailed characteristics of people with MDR/RR-TB participating in the study are shown in Table 1.

**Fig. 2:**
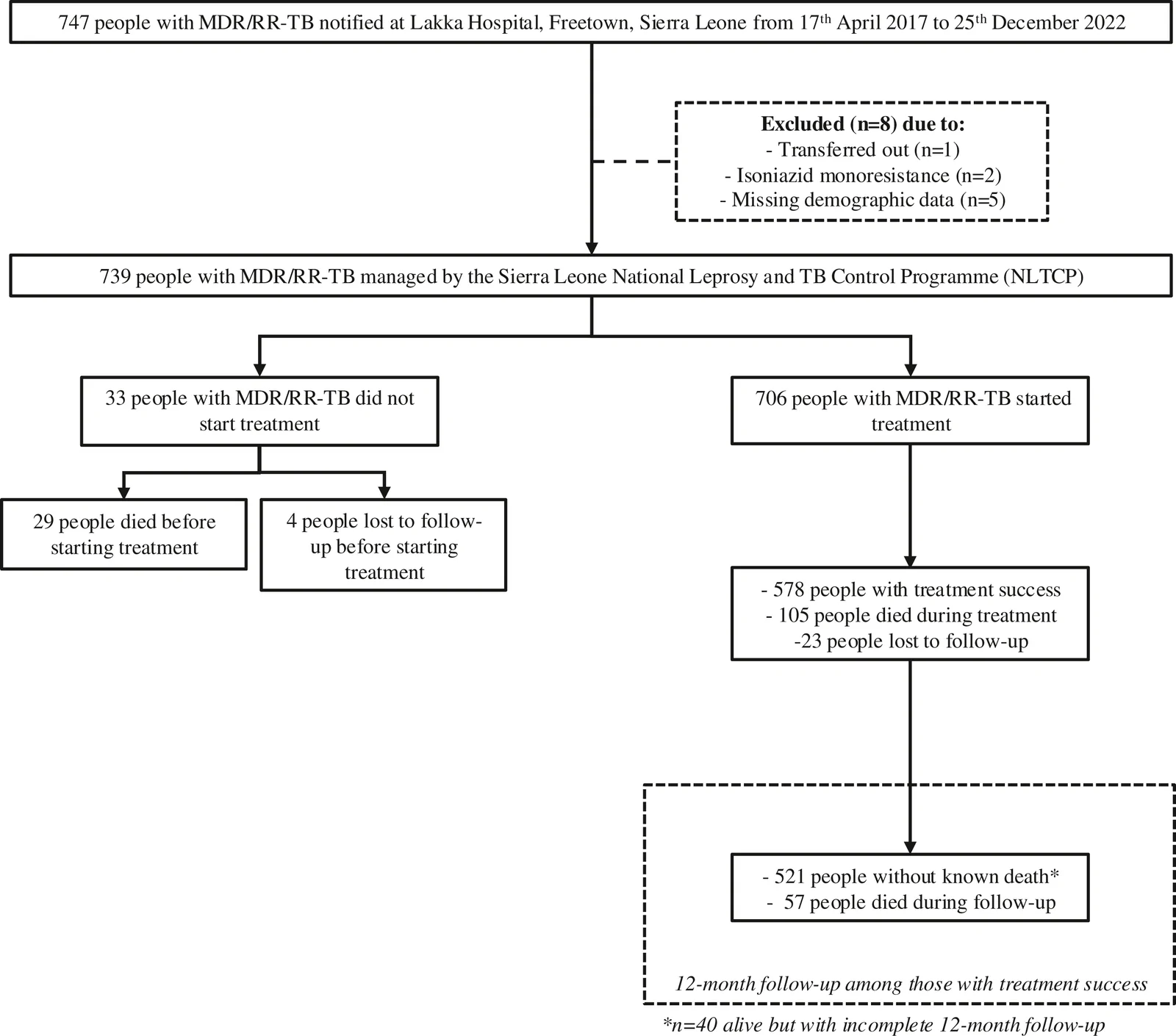
Flow diagram showing people with MDR/RR-TB included in the study. MDR/RR-TB, multidrug/rifampicin-resistant tuberculosis.

**Table 1:** Baseline characteristics of people with MDR/RR-TB in Sierra Leone participating in the study (n = 739).

	People with MDR/RR-TB (n = 739)
Demographic and social risk factors	
Age	
0–24 years^a^	154 (21%)
25–44 years	398 (54%)
45 years and over^b^	187 (25%)
Sex assigned at birth	
Male	517 (70%)
Female	222 (30%)
District residence	
Makeni Government Hospital (Northern)	194 (26%)
Bo Regional Hospital (Southern)	61 (8.3%)
Kenema Government Hospital (Eastern)	55 (7.4%)
Lakka (Western and National)	429 (58%)
History of incarceration (n = 728)	
No	707 (97%)
Yes	21 (2.9%)
Occupation prior to diagnosis (n = 736)	
Unemployed	82 (11%)
Employed	654 (89%)
Health-related risk factors	
Smoking (n = 731)	
Never smoked	528 (72%)
Smoked for less than 10 years	85 (12%)
Smoked for 10 years or more	118 (16%)
HIV (n = 734)	
HIV negative	574 (78%)
HIV positive and on ART at TB diagnosis	125 (17%)
HIV positive and not on ART at TB diagnosis	35 (4.8%)
Diabetes (n = 735)	
No	719 (98%)
Yes	16 (2.2%)
Other comorbidities (n = 732)^c^	
No	708 (97%)
Yes	24 (3.3%)
Renal impairment (n = 732)	
No	715 (98%)
Yes	17 (2.3%)
Previous TB (n = 735)	
Primary/new (ie. no previous TB)	165 (22%)
Previous TB treatment failure	368 (50%)
Relapse after previous cure/treatment success	113 (15%)
Previous LTFU on TB treatment	89 (12%)
Chronic lung disease (n = 718)	
No	631 (88%)
Yes	87 (12%)
Disease-related risk factors	
Mycobacterial culture result (n = 710)	
M.tb	452 (64%)
M.africanum	153 (22%)
Mixed/other culture result	27 (3.8%)
No growth	68 (9.6%)
Contaminated/indeterminate	10 (1.4%)
Phenotypic drug resistance	
Pyrazinamide resistance (n = 725)	
No (tested and not resistant)	616 (85%)
Yes	109 (15%)
Ethambutol resistance (n = 723)	
No (tested and not resistant)	661 (91%)
Yes	62 (8.6%)
Para-aminosalicylic acid resistance (n = 723)	
No (tested and not resistant)	710 (985)
Yes	13 (1.8%)
Prothionamide resistance (n = 722)	
No (tested and not resistant)	687 (95%)
Yes	35 (4.9%)
Capreomycin resistance (n = 723)	
No (tested and not resistant)	712 (98%)
Yes	11 (1.5%)
Sputum smear grade (n = 724)	
Negative	64 (8.8%)
Scanty (+/−)	92 (13%)
Positive +	193 (27%)
Positive ++	187 (26%)
Positive +++	188 (26%)
Chest radiography abnormalities	
Severe TB (n = 717) (cavitations or miliary)	
No	566 (79%)
Yes	151 (21%)
Miliary (n = 720)	
No	710 (99%)
Yes	10 (1.4%)
Cavitations (n = 718)	
No	574 (80%)
Yes	144 (20%)
Consolidation (n = 720)	
No	468 (65%)
Yes	252 (35%)
Pneumothorax (n = 718)	
No	691 (96%)
Yes	27 (3.8%)
Effusion (n = 717)	
No	639 (89%)
Yes	78 (11%)
Lymphadenopathy (n = 716)	
No	655 (91%)
Yes	61 (8.5%)
Nodules (n = 721)	
No	676 (94%)
Yes	45 (6.2%)
BMI (kg/m^2^) (n = 729)	
<16.5 (Severely underweight)	226 (31%)
16.5–18.4 (Underweight)	213 (29%)
18.5–24.9 (Normal weight)	258 (35%)
25.0 and over (Overweight)	32 (4.4%)
Treatment regimen	
Never started treatment	33 (4.5%)
Short	512 (69%)
Long^d^	194 (26%)

Data are n (%). For percentages, the denominator is considered as individuals with data available (stated in brackets after each variable if not 739). Formal ethnicity data was not collected but the predominant ethnicity of people treated at Lakka hospital and its environs is Black African.

MDR/RR-TB, multidrug/rifampicin-resistant tuberculosis; BMI, body mass index; LTFU, loss to follow-up.

a 0–24 years includes: 0–14 years (10, 1.4%) and 15–24 years (144, 19%).

b 45 years and over includes: 45–64 years (172, 23%) and 65 years and over (15, 2.0%).

c Any other comorbidity reported by participants or physicians excluding HIV, renal impairment, or diabetes. Documented comorbidities included hepatitis, malnutrition, sickle cell disease, and hypertension.

d Long treatment regimen encompasses both those initiated on the long regimen at treatment initiation and those switched from assigned short regimen to long following treatment failure (n = 4/739).

MDR/RR-TB treatment was started by 96% (706/739) of people, with 73% (512/706) receiving only a short regimen and 27% (194/706) a long regimen. Bedaquiline was recorded as being used for 42% (217/512) of participants who received only a short regimen, and 86% (167/194) of those on a long regimen.

### Adverse treatment outcome

Overall, 22% (161/739) of people with MDR/RR-TB had an adverse treatment outcome—18% (134/739) died (22% [29/134] before treatment, and 78% [105/134] during treatment), and 3.7% (27/739) were lost to follow-up (15% [4/27] before treatment, and 85% [23/27] during treatment). The remainder (78%, 578/739) had treatment success. 0.5% (4/739) of people were switched from a short to long regimen due to treatment failure and had their long regimen outcomes recorded. Treatment failure was not recorded for any people who received the long regimen.

Baseline demographic and social risk factors (Table 2) associated with adverse treatment outcome were being aged 45 years and over compared to 25–44 years (aOR = 1.9, 95% CI: 1.1–3.2) and unemployment (aOR = 2.9, 95% CI: 1.8–4.8).

**Table 2:** Demographic and social risk factors and their associations with adverse treatment outcome and post-treatment mortality among people with MDR/RR-TB in Sierra Leone (n = 739).

	Adverse treatment outcome (n = 739)					Post-treatment mortality (n = 578)					Adjustment set
	People with no adverse treatment outcome (n = 578)	People with an adverse treatment outcome (n = 161)	aOR	95% CI	p value	People without known death (n = 521)	People who died during 12-month follow-up (n = 57)	aOR	95% CI	p value	
Age											Sex, district residence (as competing exposures, not confounders)
0–24 years	127 (83%)	27 (18%)	Ref	–	–	118 (93%)	9 (7.1%)	–	–	–	
25–44 years	318 (80%)	80 (20%)	1.2	0.73–1.9	0.495	287 (90%)	31 (9.8%)	1.4	0.62–3.0	0.447	
45 years and over	133 (71%)	54 (29%)	1.9	1.1–3.2	0.019	116 (87%)	17 (13%)	1.8	0.74–4.2	0.200	
Sex assigned at birth											Age, district residence (as competing exposures, not confounders)
Female	171 (77%)	51 (23%)	Ref	–	–	157 (92%)	14 (8.2%)	–	–	–	
Male	407 (79%)	110 (21%)	0.84	0.57–1.2	0.380	364 (89%)	43 (11%)	1.2	0.65–2.3	0.519	
District residence											Age, sex (as competing exposures, not confounders)
Lakka (Western and National)	344 (80%)	85 (20%)	Ref	–	–	312 (91%)	32 (9.3%)	–	–	–	
Makeni Government Hospital (Northern)	146 (75%)	48 (25%)	1.3	0.86–1.9	0.226	129 (88%)	17 (12%)	1.2	0.64–2.3	0.559	
Bo Regional Hospital (Southern)	45 (74%)	16 (26%)	1.4	0.72–2.5	0.351	38 (84%)	7 (16%)	1.6	0.65–3.9	0.312	
Kenema Government Hospital (Eastern)	43 (78%)	12 (22%)	1.1	0.56–2.2	0.752	42 (98%)	1 (2.3%)	0.22	0.03–1.7	0.143	
History of incarceration											Age, sex, district residence
No	551 (78%)	156 (22%)	Ref	–	–	497 (90%)	54 (9.8%)	–	–	–	
Yes	18 (86%)	3 (14%)	0.56	0.16–1.9	0.362	16 (89%)	2 (11%)	1.2	0.26–5.4	0.828	
Occupation prior to diagnosis											Age, sex, district residence, incarceration
Employed	528 (81%)	126 (19%)	Ref	–	–	477 (90%)	51 (9.7%)	–	–	–	
Unemployed	50 (61%)	32 (39%)	2.9	1.8–4.8	<0.001	44 (88%)	6 (12%)	1.2	0.50–3.1	0.652	

MDR/RR-TB, multidrug/rifampicin-resistant tuberculosis; aOR, adjusted odds ratio; 95% CI, 95% confidence interval.

Analysis used imputed datasets with minimum adjustment sets defined by the directed acyclic graph (Fig. 1).

Baseline health-related risk factors (Table 3) included being HIV positive (aOR = 1.8, 95% CI: 1.1–2.7). When disaggregating by antiretroviral therapy (ART) status, not being on ART at TB diagnosis increased the odds of adverse treatment outcome four-fold (aOR = 4.4, 95% CI: 2.1–9.1) compared to being HIV negative, but people with HIV who were already on ART at TB diagnosis had similar odds of adverse treatment outcome compared to being HIV negative (aOR = 1.3, 95% CI: 0.8–2.1). Additional health-related risk factors were renal impairment (aOR = 4.8, 95% CI: 1.8–13); chronic lung disease (aOR = 1.7, 95% CI: 1.0–2.9); and other comorbidities (aOR = 2.8, 95% CI: 1.2–6.8).

**Table 3:** Health-related risk factors and their associations with adverse treatment outcome and post-treatment mortality among people with MDR/RR-TB in Sierra Leone (n = 739).

	Adverse treatment outcome (n = 739)					Post-treatment mortality (n = 578)					Adjustment set
	People with no adverse treatment outcome (n = 578)	People with an adverse treatment outcome (n = 161)	aOR	95% CI	p value	People without known death (n = 521)	People who died during 12-month follow-up (n = 57)	aOR	95% CI	p value	
Smoking											All demographic and social risk factors
Never smoked	413 (78%)	115 (22%)	Ref	–	–	318 (92%)	32 (7.8%)	–	–	–	
Smoked for less than 10 years	72 (85%)	13 (15%)	0.79	0.42–1.5	0.485	64 (89%)	8 (11%)	1.5	0.65–3.6	0.331	
Smoked for 10 years or more	93 (79%)	25 (21%)	0.89	0.51–1.5	0.672	76 (82%)	17 (18%)	2.7	1.3–5.5	0.007	
HIV^a^											All demographic and social risk factors
Negative	464 (81%)	110 (19%)	Ref	–	–	423 (91%)	41 (8.9%)	–	–	–	
Positive	114 (71%)	46 (29%)	1.8	1.1–2.7	0.01	98 (86%)	16 (14%)	2.0	1.0–3.9	0.047	
Diabetes											All demographic and social risk factors
No	567 (79%)	152 (21%)	Ref	–	–	511 (90%)	56 (9.9%)	–	–	–	
Yes	11 (69%)	5 (31%)	1.7	0.55–5.0	0.369	10 (91%)	1 (9.1%)	0.86	0.11–6.9	0.884	
Renal impairment											All demographic and social risk factors, diabetes, HIV, smoking
No	570 (80%)	145 (20%)	Ref	–	–	514 (90%)	56 (9.8%)	–	–	–	
Yes	8 (47%)	9 (53%)	4.8	1.8–13	0.002	7 (88%)	1 (13%)	0.98	0.11–9.0	0.989	
Other comorbidities^b^											All demographic and social risk factors, diabetes, HIV, smoking
No	564 (80%)	144 (20%)	Ref	–	–	508 (90%)	56 (9.9%)	–	–	–	
Yes	14 (58%)	10 (42%)	2.8	1.2–6.8	0.019	13 (93%)	1 (7.1%)	0.61	0.07–5.0	0.642	
Previous TB											All demographic and social risk factors, diabetes, HIV, smoking, other comorbidities, renal impairment
Primary/new	132 (80%)	23 (20%)	Ref	–	–	125 (95%)	7 (5.3%)	–	–	–	
Previous TB treatment failure	280 (76%)	88 (24%)	1.4	0.85–2.2	0.196	249 (89%)	31 (11%)	2.3	0.95–5.6	0.066	
Relapse after previous cure/treatment success	89 (79%)	24 (21%)	1.1	0.57–1.9	0.887	82 (92%)	7 (7.9%)	1.4	0.45–4.1	0.581	
Previous LTFU on TB treatment	75 (84%)	14 (16%)	0.66	0.32–1.4	0.274	63 (84%)	12 (16%)	3.1	1.1–8.7	0.032	
Chronic lung disease											All demographic and social risk factors, HIV, smoking, previous TB
No	511 (81%)	120 (19%)	Ref	–	–	467 (91%)	44 (8.7%)	–	–	–	
Yes	65 (75%)	22 (25%)	1.7	1.0–2.9	0.042	53 (82%)	12 (18%)	2.2	1.1–4.7	0.038	

MDR/RR-TB, multidrug/rifampicin-resistant tuberculosis; aOR, adjusted odds ratio; 95% CI, 95% confidence interval; TB, tuberculosis; LTFU, loss to follow-up.

Analysis used imputed datasets with minimum adjustment sets defined by the directed acyclic graph (Fig. 1).

a Timing of antiretroviral therapy (ART) initiation for individuals with HIV was disaggregated. Adverse treatment outcomes were not associated with being HIV positive on ART at TB diagnosis (aOR = 1.3, 95% CI: 0.8–2.1, p = 0.359), but were associated with being HIV positive and not on ART at TB diagnosis (aOR = 4.4, 95% CI: 2.1–9.1, p < 0.001). Post-treatment mortality was associated with being HIV positive on ART at TB diagnosis (aOR 2.0, 95% CI: 1.0–4.0, p = 0.054), but not statistically with being HIV positive and not on ART at TB diagnosis (aOR = 1.9, 95% CI: 0.4–9.0, p = 0.427).

b Any other comorbidity reported by participants or physicians excluding HIV, renal impairment, or diabetes. Documented comorbidities included hepatitis, malnutrition, sickle cell disease, and hypertension.

Disease-related risk factors (Table 4) associated with adverse treatment outcome were severely underweight (BMI < 16.5 kg/m^2^) (aOR = 1.9, 95% CI: 1.2–3.1) compared to normal weight (BMI: 18.5–25.0 kg/m^2^); receiving a long regimen (aOR = 2.0, 95% CI: 1.3–3.2) compared to only a short regimen; and having specific chest radiography abnormalities of consolidation (aOR = 1.6, 95% CI: 1.1–2.4), pneumothorax (aOR = 3.2, 95% CI: 1.4–7.5), and effusion (aOR = 1.8, 95% CI: 1.0–3.1). Having severe TB (cavitations or miliary) on chest radiography showed some evidence of association with adverse treatment outcome (aOR = 1.5, 95% CI: 0.95–2.4).

**Table 4:** Disease-related risk factors and their associations with adverse treatment outcome and post-treatment mortality among people with MDR/RR-TB in Sierra Leone (n = 739).

	Adverse treatment outcome (n = 739)					Post-treatment mortality (n = 578)					Adjustment set
	People with no adverse treatment outcome (n = 578)	People with an adverse treatment outcome (n = 161)	aOR	95% CI	p value	People without known death (n = 521)	People who died during 12-month follow-up (n = 57)	aOR	95% CI	p value	
Mycobacterial culture result											All demographic and social risk factors, all health-related risk factors
M.tb	372 (82%)	80 (18%)	Ref	–	–	337 (91%)	35 (9.4%)	–	–	–	
M.africanum	124 (81%)	29 (19%)	1.1	0.64–1.9	0.714	110 (89%)	14 (11%)	1.0	0.50–2.0	0.988	
Mixed/other culture result	20 (74%)	7 (26%)	1.5	0.53–4.4	0.431	19 (95%)	1 (5.0%)	0.37	0.05–3.0	0.355	
No growth	54 (79%)	14 (21%)	1.7	0.88–3.2	0.115	48 (89%)	6 (11%)	0.87	0.33–2.3	0.784	
Contaminated/indeterminate	7 (70%)	3 (30%)	1.5	0.35–6.4	0.595	6 (86%)	1 (14%)	1.0	0.11–9.5	0.989	
Pyrazinamide resistance						NA^a^					All demographic and social risk factors, all health-related risk factors
No (tested and not resistant)	486 (79%)	130 (21%)	Ref	–	–						
Yes	88 (81%)	21 (19%)	0.95	0.54–1.7	0.855						
Ethambutol resistance											
No (tested and not resistant)	523 (79%)	138 (21%)	Ref	–	–						
Yes	50 (81%)	12 (19%)	0.79	0.38–1.7	0.532						
Para-aminosalicylic acid resistance											
No (tested and not resistant)	565 (80%)	145 (20%)	Ref	–	–						
Yes	9 (69%)	4 (31%)	1.7	0.46–5.9	0.442						
Prothionamide resistance											
No (tested and not resistant)	546 (79%)	141 (21%)	Ref	–	–						
Yes	27 (77%)	8 (23%)	1.1	0.43–2.9	0.806						
Capreomycin resistance											
No (tested and not resistant)	566 (79%)	146 (21%)	Ref	–	–						
Yes	8 (72%)	3 (27%)	2.1	0.47–9.4	0.330						
Sputum smear grade											All demographic and social risk factors, all health-related risk factors, drug resistance
Negative	51 (80%)	13 (20%)	Ref	–	–	47 (92%)	4 (7.8%)	–	–	–	
Scanty (+/−)	66 (72%)	26 (28%)	1.7	0.79–3.9	0.172	60 (91%)	6 (9.1%)	1.3	0.31–5.2	0.751	
Positive +	157 (81%)	36 (19%)	0.98	0.47–2.1	0.962	139 (89%)	18 (11%)	1.6	0.49–5.3	0.431	
Positive ++	156 (83%)	31 (17%)	0.86	0.41–1.8	0.693	140 (90%)	16 (10%)	1.4	0.40–4.7	0.614	
Positive +++	147 (78%)	41 (22%)	1.1	0.54–2.4	0.729	134 (91%)	13 (8.8%)	1.2	0.32–4.3	0.749	
Chest radiography abnormalities											
Severe TB (cavitations or miliary)											All demographic and social risk factors, all health-related risk factors, drug resistance
No	463 (82%)	103 (18%)	Ref	–	–	428 (92%)	35 (7.6%)	–	–	–	
Yes	113 (75%)	38 (25%)	1.5	0.95–2.4	0.082	91 (81%)	22 (19%)	2.4	1.2–4.5	0.009	
Miliary											
No	570 (80%)	140 (20%)	Ref	–	–	513 (90%)	57 (10%)	–	–	–	
Yes	7 (70%)	3 (30%)	2.2	0.47–10	0.317	7 (100%)	0 (0%)	NA	NA	NA	
Cavitations											
No	468 (82%)	106 (18%)	Ref	–	–	433 (93%)	35 (7.5%)	–	–	–	
Yes	109 (76%)	35 (24%)	1.4	0.86–2.2	0.183	87 (80%)	22 (20%)	2.6	1.3–4.9	0.004	
Consolidation											All demographic and social risk factors, all health-related risk factors, disease severity (smear and severe TB on chest radiography)
No	388 (83%)	80 (17%)	Ref	–	–	355 (91%)	33 (8.5%)	–	–	–	
Yes	189 (75%)	63 (25%)	1.6	1.1–2.4	0.028	165 (87%)	24 (13%)	1.3	0.69–2.3	0.434	
Pneumothorax											
No	559 (81%)	132 (19%)	Ref	–	–	505 (90%)	54 (9.7%)	–	–	–	
Yes	17 (63%)	10 (37%)	3.2	1.4–7.5	0.007	14 (82%)	3 (18%)	3.0	0.76–12	0.118	
Effusion											
No	519 (81%)	120 (19%)	Ref	–	–	470 (91%)	49 (9.4%)	–	–	–	
Yes	57 (73%)	21 (27%)	1.8	1.0–3.1	0.048	49 (86%)	8 (14%)	1.8	0.77–4.3	0.173	
Lymphadenopathy											
No	531 (81%)	124 (19%)	Ref	–	–	482 (91%)	49 (9.2%)	–	–	–	
Yes	45 (74%)	16 (26%)	1.8	0.90–3.4	0.097	37 (82%)	8 (18%)	2.3	0.94–5.8	0.066	
Nodules											
No	538 (80%)	138 (20%)	Ref	–	–	486 (90%)	52 (9.7%)	–	–	–	
Yes	40 (89%)	5 (11%)	0.35	0.11–1.1	0.075	35 (88%)	5 (13%)	0.79	0.26–2.4	0.693	
BMI (kg/m^2^)											All demographic and social risk factors, all health-related risk factors, disease severity (smear and severe TB on chest radiography)
<16.5 (Severely underweight)	167 (74%)	59 (26%)	1.9	1.2–3.1	0.009	149 (89%)	18 (11%)	1.2	0.60–2.5	0.579	
16.5–18.4 (Underweight)	168 (79%)	45 (21%)	1.4	0.83–2.3	0.222	153 (91%)	15 (8.9%)	0.95	0.44–2.0	0.883	
18.5–24.9 (Normal weight)	216 (84%)	42 (16%)	Ref	–	–	195 (90%)	21 (9.7%)	–	–	–	
25.0 and over (Overweight)	26 (81%)	6 (19%)	1.3	0.46–3.6	0.633	23 (88%)	3 (12%)	2.1	0.48–8.9	0.334	
Treatment regimen											All demographic and social risk factors, all health-related risk factors, drug resistance, disease severity, chest radiography abnormalities
Short	437 (85%)	75 (15%)	Ref	–	–	392 (90%)	45 (10%)	–	–	–	
Long	141 (73%)	53 (27%)	2.0	1.3–3.2	0.002	129 (91%)	12 (8.5%)	1.2	0.55–2.5	0.69	
Never started treatment	0 (0%)	33 (100%)	NA	NA	NA	NA	NA	NA	NA	NA	

MDR/RR-TB, multidrug/rifampicin-resistant tuberculosis; aOR, adjusted odds ratio; 95% CI, 95% confidence interval; TB, tuberculosis; BMI, body mass index.

Analysis used imputed datasets with minimum adjustment sets defined by the directed acyclic graph (Fig. 1).

a Not analysed due to low numbers precluding meaningful interpretation.

Fig. 3 shows the PAF of adverse treatment outcomes for these risk factors. Notably, 19% (95% CI: 2.6–36) were attributable to underweight (BMI < 18.5 kg/m^2^) versus not underweight (BMI ≥ 18.5 kg/m^2^), 16% (95% CI: 5.6–27) to a long regimen; 11% (95% CI: 5.2–17) to unemployment; and 10% (95% CI: 2.0–18) to HIV.

**Fig. 3:**
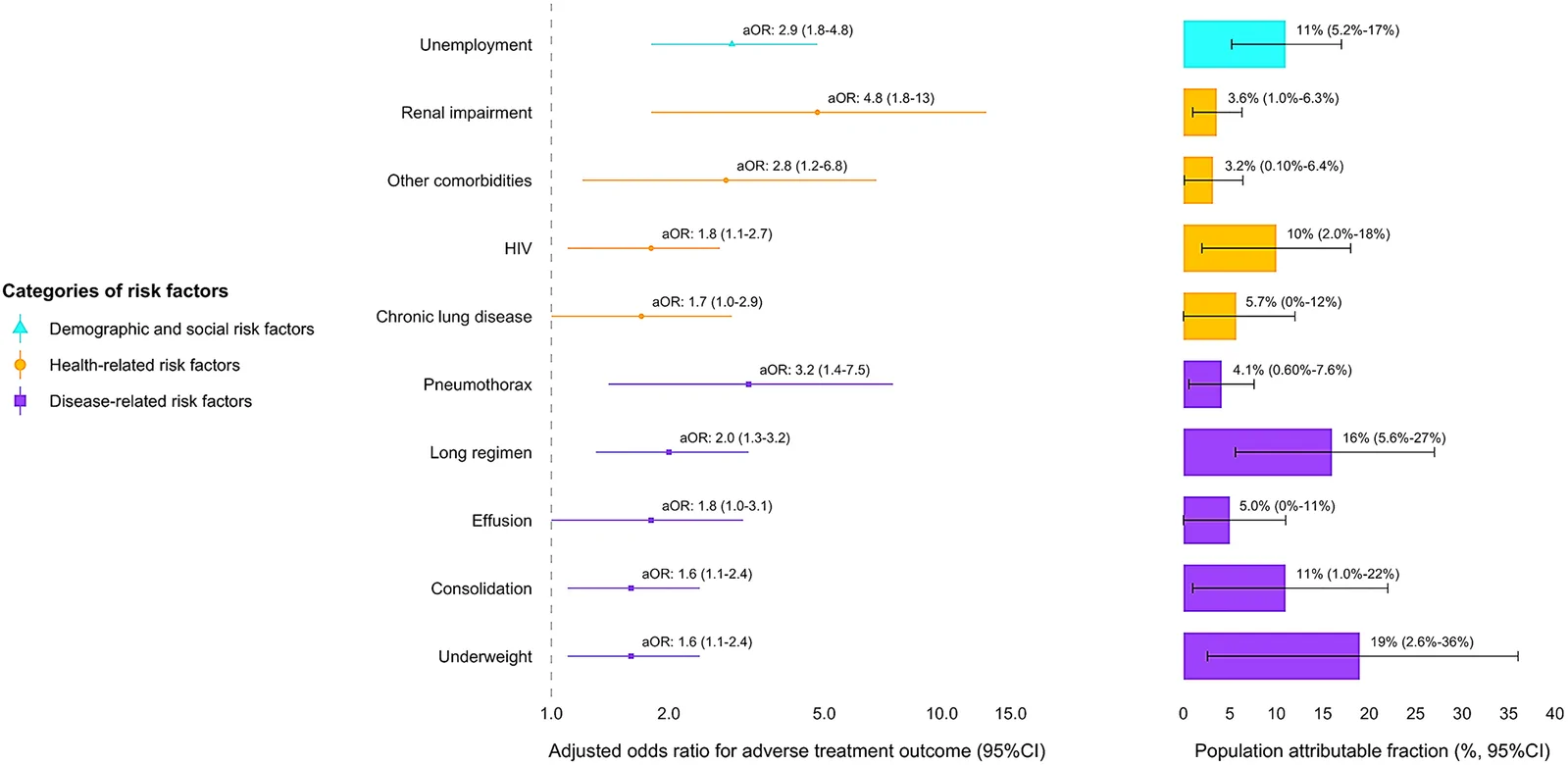
Associations and population attributable fractions for key risk factors for adverse treatment outcome among people with MDR/RR-TB in Sierra Leone (n = 739). MDR/RR-TB, multidrug/rifampicin-resistant tuberculosis. Analysis used imputed datasets with minimum adjustment sets defined by the directed acyclic graph (Fig. 1). Error bars represent 95% confidence intervals (95%CI). For this analysis, weight was dichotomised to underweight (body mass index<18.5 kg/m^2^) versus not underweight (body mass index≥18.5 kg/m^2^).

### Post-treatment mortality

Among the 78% of the cohort who had treatment success, 9.9% (57/578) were known to have died during the 12-month follow-up period. This post-treatment mortality was associated with being HIV positive (aOR = 2.0, 95% CI: 1.0–3.9); smoking for greater than 10 years (aOR = 2.7, 95% CI: 1.3–5.5) compared to never smoking; chronic lung disease (aOR = 2.2, 95% CI: 1.1–4.7); previous loss to follow-up on TB treatment (aOR = 3.1, 95% CI: 1.1–8.7) compared to no previous TB; and severe TB on chest radiography (aOR = 2.4, 95% CI: 1.2–4.5) (Table 2, Table 3, Table 4). 6.9% (40/578) of participants had less than 12 months follow-up following treatment success.

## Discussion

In this national cohort study of 739 people with MDR/RR-TB in Sierra Leone, the largest single-country MDR/RR-TB cohort study in West Africa to date, our findings demonstrate the ongoing severe clinical and public health consequences of this disease. First, despite the availability of effective treatment, one in five people with MDR/RR-TB had an adverse treatment outcome, driven by deaths occurring before or during treatment. Second, our findings demonstrate that even among people with MDR/RR-TB who have apparent treatment success, this does not equate to continued health, with one in 10 dying in the subsequent 12 months. Taken together, over a quarter of our cohort of predominantly young adults died either before treatment, during treatment, or within a year of successfully completing treatment. We identified several potentially modifiable biomedical and social risk factors driving these adverse outcomes, which demonstrate the urgent need to develop and evaluate holistic, person-centred biosocial interventions for people with MDR/RR-TB that can enhance and broaden the care, and extend throughout and beyond TB treatment.

The pharmacological treatment of MDR/RR-TB has transformed in recent years,^2^ reflected in our cohort by the fact that the great majority of participants received a short all-oral regimen. These regimens were associated with improved outcomes compared to longer regimens even after adjusting for a range of factors including disease severity, previous TB, and resistance to several second-line drugs. However, we did not have data on specific drugs included in the regimens, nor were DST data available for a range of key drugs including bedaquiline, linezolid, and fluroquinolones, limiting our ability to investigate the effect of specific treatment strategies and resistance patterns. Nevertheless, broadly, the high proportion of adverse treatment outcomes observed in our cohort highlights the importance of rapid scale-up of shortened, simplified, bedaquiline-based regimens for people with MDR/RR-TB. Improved efficacy and tolerability of newer regimens and thus cost to those with MDR/RR-TB and health systems has been established by TB-PRACTECAL, endTB and BEAT-TB. Here, non-inferiority of 6–9-month all-oral regimens including various combinations of bedaquiline, delamanid, linezolid, levofloxacin or moxifloxacin, clofazimine and pyrazinamide compared to standard care has been repeatedly illustrated, prompting WHO 2025 treatment recommendation updates.^20^, ^21^, ^22^, ^23^, ^24^ Consequently, improved medication access is fundamental to improving MDR/RR-TB treatment outcomes in Sierra Leone.

Importantly, scale-up of such regimens should be accompanied by investment in rapid, sensitive, near-person DST for second-line drugs to detect resistance early and optimise regimen composition in people with complex resistance patterns.^25^ Such tests might have particular value for ensuring prompt initiation of treatment and reducing pre-treatment mortality and loss to follow-up, an underestimated problem in TB care and prevention. Within our cohort, pre-treatment mortality and loss to follow-up accounted for 4.5% (33/739) of people, mirroring our previous study in which 6.6% (24/365) of people died or were lost to follow-up pre-treatment. We were unable to ascertain pre-treatment mortality and loss to follow-up outside of the data collected from Lakka Hospital and so our findings may be an underestimate: a systematic review and meta-analysis showed a higher pre-treatment loss to follow-up of 18% (95% confidence interval, 13–22%) for people with TB in sub-Saharan Africa.^11^^,^^26^

Our findings demonstrate that these improved tests and treatments must be accompanied by the provision of more integrated healthcare during TB treatment. Several comorbidities at diagnosis including HIV, chronic lung disease, and renal impairment were associated with adverse treatment outcome and post-treatment mortality. This highlights the phenomenon that people affected by TB experience syndemic vulnerabilities where multiple chronic conditions cluster and interact, often driven by shared structural and social risk factors.^27^ The association between HIV and adverse treatment outcome was particularly striking, with those not on ART at TB diagnosis having four-fold increased odds of adverse outcome. While Sierra Leone has made progress in ART coverage, our findings emphasise the need for timely HIV diagnosis and early ART initiation alongside close monitoring and management of drug–drug interactions to optimise MDR/RR-TB care. In a more integrated TB service, this could be accompanied by screening and management for other chronic diseases guided by local epidemiology, socio-demography, resource availability, and the preferences of people with MDR/RR-TB. This should include smoking cessation programmes, because smoking and chronic lung diseases were important risk factors for mortality during and post-treatment in our cohort and smoking is increasing in many countries with a high TB burden.^28^

The strong association between unemployment and adverse treatment outcome in our cohort reinforces how social risk factors fundamentally shape MDR/RR-TB outcomes alongside these biomedical factors.^29^ While TB diagnosis and treatment are free in Sierra Leone, people with MDR/RR-TB face substantial indirect costs including transportation for daily treatment following hospital discharge, food, and income loss.^6^ Those without employment lack the financial buffer to absorb these costs, potentially driving treatment interruptions and loss to follow-up and perpetuating the medical poverty trap.^30^ Social protection interventions (e.g. cash transfers, health insurance) have been shown to improve outcomes for people with TB in a range of settings and should be prioritised alongside improved treatments and healthcare to reduce inequity and meet WHO End TB Strategy targets related to TB and poverty.^31^ Similarly, provision of nutritional support would also contribute to reducing inequity and improving outcomes. 60% of our cohort were underweight at diagnosis, and underweight was the largest contributor to overall population risk of adverse treatment outcomes. Nutritional interventions have been shown to be effective for improving outcomes in people with TB and are recommended by WHO.^32^ Importantly, these broader holistic interventions for people with TB are likely to have myriad social, psychological, economic and health benefits extending beyond TB.

A critical and novel finding from this study is that, even after apparent treatment success, approximately one in 10 people with MDR/RR-TB died during the subsequent 12 months. Compared to trial data on long-term outcomes—especially those primarily using per protocol analyses–this study's use of routinely collected data is likely to give a mortality rate closer to real-world context. Even then, our mortality estimate is likely to be an underestimate as we report only a crude proportion without accounting for timing of death and incomplete follow-up for some participants (<7%). This finding, that apparent cure from TB does not equate to restored health, aligns with emerging evidence from other settings, including a 2019 recent systematic review showing elevated mortality persisting years after TB treatment completion, and a 2025 study in Vietnam demonstrating the same phenomenon specifically in people with MDR/RR-TB.^7^, ^8^, ^9^ While we lacked data on causes of death, TB recurrence, or chronic respiratory compromise, our analysis identified several risk factors that suggest potential causative mechanisms.

HIV is likely to be an important driver, even among those on ART, although over two thirds of deaths following treatment success occurred in people who were HIV negative. Chronic lung disease, severe TB on chest radiography at diagnosis, previous loss to follow-up on TB treatment, and smoking were all associated with post-treatment mortality. Taken together, these highlight the potential importance of post-TB lung disease, an increasingly recognised phenomenon,^33^ and indicate a critical gap in current TB programmes that typically discharge people with TB after treatment completion with no further follow-up. Establishing post-TB care pathways, including lung function assessment, screening for chronic respiratory symptoms and TB recurrence (especially in people who have previously failed treatment or had multiple TB episodes), and linkage to chronic disease management, could address this gap and have benefits for both people with MDR/RR-TB and, by breaking transmission chains, their communities.^10^ Longitudinal cohort studies with systematic assessment of post-TB sequelae (such as the TB Sequel study)^34^ and causes of death are urgently needed to understand the mechanisms driving post-treatment mortality and inform such interventions.

Our study had other limitations. First, we could only include people who reached Lakka Hospital, thereby excluding those who died before referral, were lost before MDR/RR-TB diagnosis, or never accessed care due to limited diagnostic coverage. This selection bias may therefore have led us to underestimate the true burden of adverse outcomes due to MDR/RR-TB and could also affect risk factor estimates and PAFs. Second, some variables relied on self-reporting at diagnosis, which may be subject to recall bias, and we could not assess time-varying factors such as changes in nutritional status during treatment that might influence outcomes. Third, our post-treatment mortality analysis implicitly adjusts for treatment success, introducing selection bias. The associations between baseline risk factors and post-treatment mortality may differ in this selected population compared to all people with MDR/RR-TB who were treated, as we excluded those who died or were lost to follow-up during treatment. Relatedly, due to the programmatic nature of our data, we were only able to combine death, treatment failure, and loss to follow-up in a composite adverse outcome (in line with both national guidance and WHO definitions); and not analyse specific risk factors associated with each of these sub-outcomes. Finally, some data collection occurred during the COVID-19 pandemic and associated political unrest, which may have affected both service delivery and follow-up completeness. Despite these limitations, our study represents the largest MDR/RR-TB cohort study from West Africa and is the first study from Africa to systematically assess post-treatment mortality in this population. The use of a DAG strengthens our ability to identify modifiable risk factors beyond standard, simple predictive multivariable models.^16^^,^^18^ Our findings provide actionable evidence for the Sierra Leone NLTCP and similar high-burden settings in West Africa to design comprehensive interventions addressing the full spectrum of MDR/RR-TB care from diagnosis to post-treatment follow-up.

In conclusion, our study demonstrates that MDR/RR-TB in Sierra Leone continues to be associated with adverse outcomes, undermining efforts to end the TB epidemic. Our findings suggest that achieving meaningful improvements in MDR/RR-TB care requires holistic, person-centred biosocial interventions extending throughout and beyond the treatment period that combine optimised tests and treatment regimens with integrated healthcare and social protection. Long-term, strategic, multisectoral commitment and funding will be pivotal to deliver such interventions and improve the lives of people with MDR/RR-TB globally.

## Contributors

All authors participated in the research, had access to the original dataset, and contributed to the preparation of the manuscript. The study was conceived by RFK, MJS, and TW. The data was collected by RFK, OC, LF, and RH. The data was analysed by FG, TW and MJS. The final analysed dataset was accessed, interpreted, and verified as in the reported paper by RFK, FG, MJS, and TW. The first draft of the manuscript was written by FG and MJS. The manuscript was reviewed, finalised, and approved as submitted by all authors who all take responsibility for its publication.

## Data sharing statement

Requests for the data used in this study can be made via the corresponding author.

## Declaration of interests

All authors declare that they have no conflict of interest in relation to this research.

## Appendix A Supplementary data

Supplementary Methods

EPD form
