# Supplementary material for: Treatment outcomes and post-treatment mortality among people with multidrug/rifampicin-resistant tuberculosis in Sierra Leone: a national cohort study
Source: Lancet Reg Health Afr. 2026 Oct;8:None. doi: 10.1016/j.lanafr.2026.100061 (PMC13672282; doi:10.1016/j.lanafr.2026.100061)
Supplement: Supplementary file 1 [file mmc2.pdf]

Equitable Partnership Declaration

If any questions do not apply to your study, please indicate "N/A" for "not applicable.
For more information on how to complete this form see the Information for Authors document.

Researcher considerations

1. Please detail the involvement that researchers who are based in the country or countries of study had during a) study design; b) clinical study processes, such as processing blood samples, prescribing medication, or patient recruitment; c) data interpretation; and d) manuscript preparation, commenting on all aspects. If they were not involved in any of these aspects, please explain why.

This should include a thorough description of their leadership roles in the study. Are local researchers named in the author list or the acknowledgements, or are they not mentioned at all (and, if not, why)? Please also describe the involvement of early career researchers based in the location of the study. Some of this information might be repeated from the Contributors section in the manuscript. Note: we adhere to ICMJE authorship criteria for naming authors on a paper.

a) Study design:
Clinical researchers in Sierra Leone designed data collection tools and collated data during routine programmatic activities. The study design of a retrospective cohort with adverse treatment outcome as the key outcome variable was previously designed collaboratively between Dr Rashidatu Fouad Kamara (lead clinical researcher, Sierra Leone, former head of DR-TB Programme)) and Dr Tom Wingfield (UK) for our previous related study published in The Lancet Global Health.

Dr Kamara and Dr Wingfield then invited Dr Saunders and Ms Greenwood, two UK-based clinical researchers, to lead analysis, co-lead interpretation, and lead manuscript. The team also considered together how best to incorporate programmatic post-treatment success follow-up into the study and, collectively, agreed on the post-treatment success mortality outcome measure. The analysis design was co-developed across the research team and carried out by Ms Greenwood with direct supervision from Dr Saunders. This structure was agreed with Dr Wingfield and Dr Kamara based on availability and distribution of expertise.

The team's collaboration together now extends over five years and has produced another well-cited publication.

b) Clinical study processes:
The data collection was led by Dr Kamara. All data was collected and initially cleaned and collated by the Sierra Leone partners and DR-TB programme healthcare workers including Dr Ronnie Harding, Dr Ousman Conteh and Dr Lynda Foray, all co-authors on the paper, as part of routine DR-TB programme data collection. Follow-up was also conducted by these same colleagues and their field teams who did not meet the eligibility for co-authorship but were thanked formally in the acknowledgements section.

**c) Data interpretation:**<br>Dr Kamara, Dr Wingfield, Dr Saunders, and Ms Greenwood met regularly during the data analysis period to contextualise and interpret findings. Outside of these meetings, the team corresponded by email, agreeing interpretation through consensus including about issues that were complex such as the impact of adverse drug reactions or treatment regimen on outcome measures. Dr Harding, Dr Conteh, and Dr Foray also inputted into interpretation of data once packaged by the other authors.
**d) Manuscript preparation:**<br>Dr Kamara contributed to the writing of the introduction and discussion, including through considering and extracting useful themes and literature from her previous first-author publication with the team in The Lancet Global Health.<br><br>Ms Greenwood led the writing of the initial draft of the manuscript with support, especially relating to writing of statistical findings and analytical outputs, by Dr Saunders. Dr Kamara inputted into vital elements of the Methods section and supplementary file of the manuscript including especially the health system and DR-TB programmatic structure in Sierra Leone, DR-TB care and treatment regimens (and changes over time), and the DR-TB team's programmatic activities during and beyond treatment.<br><br>Dr Wingfield, Dr Kamara, Ms Greenwood, and Dr Saunders, all collectively inputted into the discussion section of the manuscript, including cross-referencing our previous Lancet Global Health paper findings with the new paper's findings.<br><br>Dr Saunders with supervision from Dr Wingfield led the response letter and updates to the manuscript following original review by reviewers of The Lancet Infectious Diseases.<br><br>All authors (Dr Kamara, Dr Wingfield, Dr Saunders, Ms Greenwood, Dr Conteh, Dr Harding, and Dr Foray) reviewed the final version of the manuscript.

2. How was funding used to remunerate and enhance the skills of researchers in the countries of study? And how was funding used to improve research infrastructure at the study sites?

   Potentially effective investments into long-term skills and opportunities within local institutions could include training or mentorship in analytical techniques and manuscript writing, opportunities to lead all or specific aspects of the study, financial remuneration rather than requiring volunteers, and other professional development and educational opportunities.

   Improvements to research infrastructure could include funding extended trial designs (eg, platform trials), establishment of long-term contracts for research staff, building research facilities, and setting up local control of funding allocation.

**Skills:**<br>Dr Wingfield worked with Dr Kamara without funding to support and develop her skills in data collation, cleaning, analysis, and manuscript writing. The whole team worked together to improve our skills in manuscript writing.
**Research infrastructure:**

Dr Wingfield and Dr Kamara collaborated on and successfully were awarded a Royal Society of Tropical Medicine and Hygiene ECR award to Dr Kamara, which was based on some of this work.

We used a buddying system for this research. Dr Saunders buddied with Ms Greenwood to support the analysis of the cohort study and facilitate Ms Greenwood's development in this area. Dr Kamara and Dr Wingfield took on the role of supervisors for the overall project with Dr Kamara focusing on supporting details of the research setting, population, and context, plus providing Ms Greenwood with her experience and feedback having previously led the first national DR-TB retrospective cohort study previously; and Dr Wingfield leading on how to adapt MSc (Greenwood) and PhD (Kamara) theses' chapters/sections, both of which used some of the findings presented in this paper, into a publishable work for peer review.

Dr Wingfield has supported Dr Kamara directly over the last five years to develop her skills in manuscript writing, thesis development, and grant writing and delivery. Dr Kamara has provided Dr Wingfield with knowledge and skills in how to design data collection tools and activities that are suitable both for practical use in the field programmatically and also robust enough to support research activities.

3. How did you safeguard the researchers who implemented the study?

Please describe how you guaranteed safe working conditions for study staff, including provision of appropriate personal protective equipment, protection from violence, and prevention of overworking.

Followed DR-TB programme policies and practices. Allowed time for holidays, time off and promoted equitable and comfortable living and working conditions.

Benefits to the communities and regions of study

4. How does the study address the research and policy priorities of its location?

How were the local priorities determined and then used to inform the research question? Who decided which priorities to take forward? Which elements of the study address those priorities?

DR-TB is a public health emergency in Sierra Leone. The Sierra Leonean Clinical Researchers on this paper are all involved in the DR-TB response and the results from our previous and current research are being translated into policy and practice change through engagement with key stakeholders.

5. How will research products be shared in the community of study?

For instance, will you be providing written or oral layperson summaries for non-academic information sharing? Will study data be made available to institutions in the region(s) of study? The Lancet Global Health *encourages authors to translate the summary (abstract) into relevant languages after paper editing; do you intend to translate your summary?*

We will aim to do another Lancet podcast as we did for our previous research. Dr Kamara has conducted a national stakeholder workshop in Sierra Leone, which involved the other Sierra Leonean co-authors (Harding, Conteh, Foray) and was supported, in grant writing and workshop design, by Dr Wingfield, to consider the perspectives of people with TB and other key stakeholders including DR-TB Programme Staff and policy makers. Dr Kamara and these co-authors plan to work with the wider DR-TB team and selected stakeholders who attended the workshop to develop policy briefs and community engagement pamphlets about the research findings.

6. How were individuals, communities, and environments protected from harm?

a) *How did you ensure that sensitive patient data were handled safely and respectfully? Was there any potential for stigma or discrimination against participants arising from any of the procedures or outcomes of the study?*

The data collected was part of routine programmatic data collection of people admitted to Lakka Hospital on treatment for DR-TB. Patients were treated respectfully, informed consent taken, and the healthcare worker team have worked hard to put in place safeguards against stigmatising or discriminatory behaviour.

b) *Might any of the tests be experienced as invasive or culturally insensitive?*

No, all were tests that were part of routine DR-TB care.

c) *How did you determine that work was sensitive to traditions, restrictions, and considerations of all cultural and religious groups in the study population?*

Dr Kamara has developed a team that has received personal and formal training in holistic person-centred care that ensures sensitivity to diverse cultures/traditions/religions. This concept of equity is also enshrined in national policy and practice documents in Sierra Leone.

All study data collection tools and plans for data collection were subject to review amongst the leadership team of the DR-TB programme, who rigorously screened the contents to ensure sensitivity of the questions included and data collected, and maintain confidentiality and anonymity. This was also reinforced by wider training for healthcare workers in the DR-TB programme on involving people under their care in data collection, within and without a research context.

d) *Were biowaste and radioactive waste disposed of in accordance with local laws?*

Not applicable

e) *Were any structures built that would have impacted members of the community or the environment (such as handwashing facilities in a public space)? If so, how did you ensure that you had appropriate community buy-in?*

Not applicable

f) *How might the study have impacted existing health-care resources (such as staff workloads, use of equipment that is typically employed elsewhere, or reallocation of public funds)?*

Data collection was part of routine data collection as part of DR-TB Programme activities. Sierra Leonean staff members and team members were not asked to do additional activities.

7. Confirm that local ethics review was sought, and please provide the approval number. If not sought, please explain why.

Local ethical approval was sought and granted from Sierra Leone Ethics and Scientific Review Committee: SLESRC No: 015/11/2023

Secondary analyses

8. Have the data analysed in your study been extracted from another source, such as a national survey, rather than being directly collected by the authors of this paper?

No

If the authors of this paper were not involved in data collection, how were the findings interpreted with sufficient contextual knowledge?
The Lancet Global Health *believe contextual understanding is crucial for informed data analysis and interpretation.*

Not applicable. Sierra Leone-based authors collected the data.

---

9. Please provide the title (eg, Dr/Prof, Mr/Mrs/Ms/Mx), name, and email address of an author who can be contacted about this statement.

Name: Dr Rashidatu Fouad Kamara // Dr Tom Wingfield
Email: shidakay700@gmail.com // **tom.wingfield@lstmed.ac.uk**

10. Finally, please provide the title and name of an author from one country of study who has seen and approved this form.

**Name:** Dr Rashidatu Fouad Kamara
