# Supplementary material for: Treatment outcomes and post-treatment mortality among people with multidrug/rifampicin-resistant tuberculosis in Sierra Leone: a national cohort study
Source: Lancet Reg Health Afr. 2026 Oct;8:None. doi: 10.1016/j.lanafr.2026.100061 (PMC13672282; doi:10.1016/j.lanafr.2026.100061)
Supplement: Supplementary file 2 [file mmc1.docx]

**Treatment outcomes and post-treatment mortality among people with multi-drug/rifampicin-resistant tuberculosis in Sierra Leone: a national cohort study (supplementary appendix)**

Supplementary methods	2

Treatment regimens	3

MDR/RR-TB outcomes	4

Treatment categories	5

References	6

#### Supplementary methods

**Microbiological testing**

In Sierra Leone, sputum samples for Xpert MTB/RIF, culture, smear microscopy for acid-alcohol fast bacilli, and phenotypic drug susceptibility testing (DST) are taken at baseline for all people with tuberculosis (TB) who are identified to be at high risk for drug resistance (e.g. because of contact with someone with known drug resistance or because of previous treatment failure).

- Xpert MTB/RIF is used as the initial diagnostic test to identify *M. tuberculosis* DNA and mutations associated with rifampicin resistance. This is decentralised at lower-level laboratories enabling results to be obtained in less than two hours.
- Culture in liquid medium is the current reference standard in Sierra Leone for bacteriological confirmation and identification of causative strain, with sputum specimens sent at baseline and monthly for 11 months and results classified according to growth. Results are dependent upon good quality specimens, prompt laboratory transport, and high-quality processing. They are defined as ‘negative’, ‘*M. tuberculosis*’, ‘*M. africanum’ and* ‘Mixed/other’ comprising both *M.tuberculosis* complex organisms and non-tuberculous mycobacteria.
- Sputum samples are obtained for smear microscopy at baseline and then monthly for eight months with results reported as ‘negative’, ‘scanty (i.e. +/-)’, ‘positive +’, ‘positive ++’ and ‘positive +++’
- Sputum samples are sent to a supranational laboratory in Borstel, Germany. The time between collecting samples to sending and being received by the supranational laboratory is 1-2 weeks with a subsequent time of 2-4 weeks to obtain phenotypic DST results. Hence, the total time from samples being taken to results being issued is approximately 4-6 weeks.

In line with World Health Organization (WHO) guidelines, bacteriologically confirmed multidrug/rifampicin-resistant tuberculosis (MDR/RR-TB) cases were those with a positive baseline microscopy smear (including those with scanty bacilli), culture, or Xpert MTB/RIF.^1^ Recognising the differing strengths of each diagnostic technique, a diagnosis of MDR/RR-TB is made by the Sierra Leone National Leprosy and TB Control Programme (NLTCP) upon consideration of individual laboratory results in combination with epidemiological and clinical characteristics.

#### Sierra Leone NLTCP MDR/RR-TB Treatment regimens

These are aligned with WHO 2016 and 2019 guidelines. Some dosages adjusted for renal impairment^2^

#### MDR/RR-TB outcomes

**Table S1:** Sierra Leone NLTCP MDR/RR-TB treatment definitions

| **OUTCOME** | **DEFINITION** |
| --- | --- |
| **Treatment failure** | Termination of treatment regimen or permanent change to new regimen/treatment strategy due to no clinical and/or bacteriological response, adverse drug reactions, or evidence of additional resistance to medications in the original regimen |
| **Loss to follow-up** | Individual who did not start treatment or had treatment interrupted for 2 or more consecutive months |
| **Died** | Died for any reason before starting treatment or during treatment course* |
| **Cured** | Individual with bacteriologically confirmed pulmonary TB at treatment initiation who completed treatment as per national policy recommendations with evidence of bacteriological response (i.e. at least two consecutive cultures at least 7 days apart are negative), with no evidence of treatment failure |
| **Treatment completed** | Treatment completed as per national policy recommendations with outcome not meeting the definition of cure or treatment failure |
| **Treatment success** | The sum of those who completed treatment and those who were cured |
| **Not evaluated** | No treatment outcome assigned in NLTCP treatment register including those ‘transferred out’ to another treatment centre with unknown outcome, but excluding individuals lost to follow-up. |

Legend: Based upon WHO (2021) definitions^3^

*Only those deaths occurring before and during treatment would be documented as deaths in the outcomes recorded in the NLTCP MDR/RR-TB register, which is used to report to bodies such as WHO. Deaths recorded after treatment would not be captured in the NLTCP MDR/RR-TB register.

#### Treatment categories

**Table S2:** Sierra Leone programmatically defined treatment categories (2016 to present)

| **WHO definition^4^** | ***Current* treatment category**  **(2020 – 2024)** | **Definition** | **Empiric treatment regimen** | ***Previous* treatment category**  **(2016 to 2020)** | **Definition** | **Empiric treatment regimen** |
| --- | --- | --- | --- | --- | --- | --- |
| New | Primary/New | Resistance in people with no history of previous TB treatment or those who have received TB treatment for less than one month previously | Short | New | No previous history of TB treatment | Short |
| Previous TB treatment failure | Category 1 | Resistance in people previously treated for drug-sensitive TB | Short | Category 1 | Unsuccessful drug-sensitive TB treatment regimen for new patients; people who remain sputum-smear-positive or culture-positive for 5 months or longer during drug-sensitive TB treatment. | Short |
|  | Category 2 | Unsuccessful drug-sensitive TB re-treatment regimen | Long | Category 2 | Unsuccessful drug-sensitive TB retreatment regimen; people who are sputum-smear- positive at 5 months or later during their category 2 retreatment, which includes streptomycin in addition to the first-line drugs. | Short |
|  | Category 3 | Individuals transferred to all-oral treatment regimen after conversion failure at month six of injectable treatment regimen | Long | N/A | N/A | N/A |
|  | Category 4 | Individuals with pre-extensively drug-resistant TB | Long | N/A | N/A | N/A |
| Relapse after previous cure/treatment success | Loss to follow-up/relapse/  treatment interruption | People who were previously treated for TB and whose most recent treatment was interrupted for 2 or more consecutive months | Long | Relapse | People who were previously treated for TB, declared cured or treatment completed at the end of their most recent course of treatment, and are now diagnosed with a recurrent episode of TB (either a true relapse or a new episode of TB caused by reinfection) | Long |
| Previous loss to follow-up on TB treatment |  |  |  | LTFU | People who were previously treated for TB and whose most recent treatment was interrupted for 2 or more consecutive months | Long |

#### References

1.	WHO consolidated guidelines on tuberculosis: module 4: treatment and care. Geneva: World Health Organization, 2025.

2.	WHO consolidated guidelines on drug-resistant tuberculosis treatment. Geneva: World Health Organization, 2019.

3.	Linh NN, Viney K, Gegia M, et al. World Health Organization treatment outcome definitions for tuberculosis: 2021 update. *Eur Respir J* 2021; **58**(2): 2100804.

4.	Definitions and reporting framework for tuberculosis. Geneva: World Health Organization, 2013 revision, updated December 2014 and January 2020.
